# A global ensemble of ocean wave climate projections from CMIP5-driven models

**DOI:** 10.1038/s41597-020-0446-2

**Published:** 2020-03-27

**Authors:** Joao Morim, Claire Trenham, Mark Hemer, Xiaolan L. Wang, Nobuhito Mori, Mercè Casas-Prat, Alvaro Semedo, Tomoya Shimura, Ben Timmermans, Paula Camus, Lucy Bricheno, Lorenzo Mentaschi, Mikhail Dobrynin, Yang Feng, Li Erikson

**Affiliations:** 1 School of Built Environment and Engineering, Griffith University, Southport, Queensland Australia; 2 Commonwealth Scientific and Industrial Research Organisation (CSIRO) Oceans and Atmosphere, Hobart, Tasmania Australia; 3 Environment and Climate Change Canada, Climate Research Division, Toronto, Ontario Canada; 4 Disaster Prevention Research Institute, Kyoto University, Kyoto, Japan; 5 Department of Water Science and Engineering, IHE-Delft, Delft, The Netherlands; 6 Climate and Ecosystems Science Division, Lawrence Berkeley National Laboratory (LBNL), Berkeley, California USA; 7 Environmental Hydraulics Institute IH Cantabria, Universidad de Cantabria, Santander, Spain; 8 National Oceanographic Centre, Liverpool, United Kingdom; 9 European Commission, Joint Research Centre (JRC), Ispra, Italy; 10 Institute of Oceanography, Center for Earth System Research and Sustainability (CEN), Universität Hamburg, Hamburg, Germany; 11 US Geological Survey (USGS), Pacific Coastal and Marine Science Center, Santa Cruz, California USA

**Keywords:** Physical oceanography, Climate change, Databases

## Abstract

This dataset, produced through the Coordinated Ocean Wave Climate Project (COWCLIP) phase 2, represents the first coordinated multivariate ensemble of 21^st^ Century global wind-wave climate projections available (henceforth COWCLIP2.0). COWCLIP2.0 comprises general and extreme statistics of significant wave height (*H*_*S*_), mean wave period (*T*_*m*_), and mean wave direction (*θ*_*m*_) computed over time-slices 1979–2004 and 2081–2100, at different frequency resolutions (monthly, seasonally and annually). The full ensemble comprising 155 global wave climate simulations is obtained from ten CMIP5-based state-of-the-art wave climate studies and provides data derived from alternative wind-wave downscaling methods, and different climate-model forcing and future emissions scenarios. The data has been produced, and processed, under a specific framework for consistency and quality, and follows CMIP5 Data Reference Syntax, Directory structures, and Metadata requirements. Technical comparison of model skill against 26 years of global satellite measurements of significant wave height has been undertaken at global and regional scales. This new dataset provides support for future broad scale coastal hazard and vulnerability assessments and climate adaptation studies in many offshore and coastal engineering applications.

## Abstract (summary)

**Table:** 

Measurement(s)	surface ocean wave parameters (significant wave height, mean wave period and mean wave direction)
Technology Type(s)	computational modeling technique
Factor Type(s)	time-frame frequency • geographic location
Sample Characteristic - Environment	climate system • ocean
Sample Characteristic - Location	Earth (planet)

Machine-accessible metadata file describing the reported data: 10.6084/m9.figshare.11940576

## Background & Summary

Wind-generated waves are recognized as a key element of the climate system^1^, having considerable environmental^2,3^, geophysical^3,4^ and socioeconomic^5^ impacts globally. They are considered paramount to navigation planning, offshore and coastal engineering activities, and energy generation (from fossil to renewable energy)^6^ with structural design strongly dependent on wind-wave characteristics.

Furthermore, ocean waves are considered dominant drivers of coastal dynamics and stability^7,8^, and are key contributors to coastal sea-level extremes at multiple time-scales^9,10^. Hence, integrating non-stationary multivariate wave conditions into broad-scale comprehensive assessments of future coastal hazards and vulnerability is critical^10,11^ to avoid potentially costly maladaptation^12^. These assessments must consider not only wave run-up and swash contributions^9,10,13^ but also changes in littoral sediment supply whose effects on the open coasts can be as considerable as effects of projected future sea-level rise^13–15^. Impacts of a changing wave climate might also affect surfing tourism worldwide, a growing market with economic relevance^16^.

However, projected wave climate data is not available among the standard suite of climate variables used to characterize the climate system^1,17^ since coupled atmosphere-ocean general circulation models (GCMs) under the Coupled Model Intercomparison Project 5 (CMIP5)^18^ do not usually include wind-wave-dependent parameterizations As a result, the availability of projected wind-wave climate data is limited relative to other climatological parameters such as temperature, precipitation and/or sea level. Using atmospheric forcing derived from CMIP5 GCM models to force dynamical or statistical wave models, multiple international climate research groups^19–28^ have developed ensembles of global wave climate projections. However, these standalone studies cover different subsets of the uncertainty space (e.g., number of climate models, or emission scenarios), use different wave downscaling approaches, consider different historical and future simulation periods and provide different wave characteristics, within a range of data formats.

Hence, to date, there is no consistent global multivariate dataset of global wave climate projections capable of sufficiently sampling the uncertainty associated with projected future ocean wave climate available^29^ for widespread use by stakeholders, government, and the research community. Here, we describe the first community-driven dataset (COWCLIP2.0) of 21^st^ century global wind-wave climate projections comprising different dynamical and statistical downscaled data. This collection assembles ten individual global datasets and was created under a pre-designed sampling framework established by the Coordinated Ocean Wave Climate Project (COWCLIP)^30–32^.

The COWCLIP2.0 dataset aims to meet current needs from many different perspectives, through the provision of an open access spatial data collection which provides consistent data (in terms of format, resolution and quality) across the global ocean. This dataset archived in Network Common Data Form (NetCDF) with CF (Climate & Forecasts) compliant metadata contains a large ensemble of 148 global ocean wave climate projections gridded on a 1° spatial grid resolution (i.e., a common grid is imposed on the various resolutions of the different datasets - section 2.3.2). The dataset provides a variety of standard wave statistics for present-day and future global multivariate wave fields (*H*_*S*_, *T*_*m*_ and *θ*_*m*_) at monthly, seasonal and annual time scales (Table 1). The COWCLIP2.0 data also includes a new set of extreme *H*_*S*_ indices designed by the Expert Team on Climate Change Detection (hereafter ETCCDI)^33^ (https://www.wcrp-climate.org/data-etccdi). These represent an additional set of ocean wave statistics (Table 2) relevant to climate change detection for a range of scientific applications.

**Table 1:** Summary of the wave contributions to the COWCLIP2.0 intercomparison data set.

Research centre	CSIRO^19^	JRC^20^	USGS^21^	NOC^22^	ECCC (d)^23^	IHE^24^	LBNL^25^	KU^26^	IHC^27^	ECCC (s)^28^
Country	Australia	EU	US	UK	Canada	Netherlands	US	Japan	Spain	Canada
Emission scenario	RCP4.5/8.5	RCP4.5/8.5	RCP4.5/8.5	RCP4.5/8.5	RCP8.5	RCP8.5	RCP8.5	RCP8.5	RCP4.5/8.5	RCP4.5/8.5
Number of GCM(s) used	8	6	4	1	5	1	1	4	29	20
**Atmospheric downscaling (high-resolution atmospheric models and/or regional climate models)**										
Atmospheric downscaling	No	No	No	No	No	No	CAM5^a^	MRI-AGCM^b^	No	No
**Wind-wave modelling configuration (WMM)**										
Wind-wave modelling method	Dynamical	Dynamical	Dynamical	Dynamical	Dynamical	Dynamical	Dynamical	Dynamical	Statistical	Statistical
Statistical/Spectral wave model	WW3	WW3	WW3	WW3	WW3	WAM4.5	WW3	WW3	Weather type	Regression
Surface wind/SLP forcing^c^	3-hourly	3-hourly	3-hourly	3-hourly	3-hourly	3-hourly	3-hourly	6-hourly	Daily SLP	6-hourly SLP
Atmospheric correction	—	—	—	—	—	—	—	—	SLP	SLP
Source-term package^d^	ST3 (BJA)	ST4	ST2	ST4	ST4	ST3	ST4	ST4	—	—
Calibration	Default	Default	Default	Default	Default	Default	Default	Default	—	—
Sea-Ice forcing	Monthly	No	No	Daily	Daily	Daily	Monthly	Monthly	—	—
Spatial resolution (°)	1 × 1	1.5 × 1.5	1.25 × 1	~0.7 × 0.5	1 × 1	1 × 1	0.25 × 0.25	~0.56 × 0.56	1 × 1	1 × 1
Spectral partition	29f × 24d	25f × 24d	25f × 24d	30f × 36d	29f × 24d	32f × 24d	32f × 36d	29f × 36d	—	—
Bathymetry data	ETOPO	ETOPO	DBDB2	GEBCO	DBDB2	ETOPO	ETOPO	ETOPO	—	—

The emission scenarios (RCP pathways) and wave downscaling approaches used by each wave climate modelling group are provided. The specific GCM models used by each climate modelling group are provided in Supplementary Table 1.

^a^observed SST obtained from the HadISST1-based data set were used to force the atmospheric model CAM5.

^b^SST0 to SST3 correspond to four different SST future change patterns derived from CMIP5 GCM models to force the atmospheric model MRI-AGCM^32^.

^c^Surface wind/Sea level pressure (SLP) forcing used to drive the wave simulations.

^d^Source-term physics (e.g., whitecapping dissipation formulation) used in the spectral wave model (consistent with the definitions used in the WW3 manual).

**Table 2:** Summary of the variables and standard wave statistics included in the COWCLIP2.0 data set.

COWCLIP2.0 set of wave statistics				
Variable	Statistics ID	Indicator name	Time-frame resolutions	Units
**via** ***getStat.f***				
*H*_*S*_	Hs_avg	Mean significant wave height	Annual (1), Seasonal (4) and Monthly (12)	m
	Hs_p10	10th Percentile significant wave height	Annual (1), Seasonal (4) and Monthly (12)	m
	Hs_p50	50th Percentile significant wave height	Annual (1), Seasonal (4) and Monthly (12)	m
	Hs_p90	90th Percentile significant wave height	Annual (1), Seasonal (4) and Monthly (12)	m
	Hs_p95	95th Percentile significant wave height	Annual (1), Seasonal (4) and Monthly (12)	m
	Hs_p99	99th Percentile significant wave height	Annual (1), Seasonal (4) and Monthly (12)	m
	Hs_max	Maximum significant wave height	Annual (1), Seasonal (4) and Monthly (12)	m
*T*_*m*_	Tm_avg	Average mean wave period	Annual (1), Seasonal (4) and Monthly (12)	s
	Tm_p10	10th Percentile mean wave period	Annual (1), Seasonal (4) and Monthly (12)	s
	Tm_p50	50th Percentile mean wave period	Annual (1), Seasonal (4) and Monthly (12)	s
	Tm_p90	90th Percentile mean wave period	Annual (1), Seasonal (4) and Monthly (12)	s
	Tm_p95	95th Percentile mean wave period	Annual (1), Seasonal (4) and Monthly (12)	s
	Tm_p99	99th Percentile mean wave period	Annual (1), Seasonal (4) and Monthly (12)	s
	Tm_max	Maximum mean wave period	Annual (1), Seasonal (4) and Monthly (12)	s
**via** ***getStatDir.f***				
*θ*_*m*_	*θ*_*m*__avg	Circular mean	Annual (1), Seasonal (4) and Monthly (12)	°N
	*θ*_*m*__std	Circular standard deviation	Annual (1), Seasonal (4) and Monthly (12)	°N

The COWCLIP2.0 dataset overcomes many previous limitations^29^, including lack of standardisation amongst existing CMIP5-driven global wave field simulations (e.g. wave variables and their statistics, spatial coverage and resolution and time-slices used for simulation) and limited sampling of dominant sources of uncertainty (e.g., model forcing and wave-downscaling uncertainties). This extensive wave information can now be widely used by different research communities (e.g. those focusing on natural hazards, coastal management, renewable energy, and ship navigation). The purpose is for this dataset to expand, as further projections of future global wave climate become available. It is envisaged that open and easy access to such dataset might provide a new stimulus and facilitates broad-scale coastal hazard and vulnerability assessments. It is also a robust basis for a range of inter-comparison analyses (e.g., quantification of sources of uncertainty)^29^, given the size and diverse nature of this dataset. For instance the annual and seasonal set of wave statistics from the COWCLIP2.0 ensemble were recently used to quantify the robustness and uncertainties in multivariate global wave projections^34^.

The development of the COWCLIP2.0 dataset helps wave researchers and data users to address the previous limited sampling of dominant uncertainties (e.g., model forcing and wave-downscaling) and significantly enhances interoperability. Before this dataset was created, researchers could access only a limited range of simulations, meaning assessment across projection scenarios and intra and/or inter-model ensembles were challenging^31,35^, with little possibility of sampling the uncertainty among wave downscaling methodologies. The inconsistencies in output wave parameters and data structures made intercomparison analysis between wave data produced by different modelling groups difficult.

## Methods

In this data descriptor, we explain the methods and techniques used to generate the original data; the data acquisition process; the standardized framework applied; the methodology used to derive the vast range of wave parameters/statistics for historical and future periods; and the computational processing used to create this consistent global dataset.

The dataset presented has been compiled from ten standalone CMIP5-based global wave projection datasets, which have been extensively described elsewhere. Those wave projection data sets draw on thirty-three different CMIP5 climate models to force the dynamical and statistical wave models, listed in Table 1. In this section, we provide a concise description of the original data created by each wave climate modelling group, with the details of each contribution provided in Table 1.

### CMIP5 GCM-forced dynamical global simulations

#### CSIRO: Multiple-model multiple-scenario ensemble

Hemer and Trenham^19^ (hereafter CSIRO) developed a global wind-wave climate projection dataset derived using a dynamical wave approach. Surface wind fields (10 m) at 3-hourly temporal resolution and sea-ice fields at monthly frequency, taken from eight CMIP5 GCMs, were used to drive a global WAVEWATCH III (WW3)^36^ wave model at 1° spatial grid resolution. The WW3 was setup using the ST3 (BAJ) source-term physics. The simulations were conducted under RCP4.5 and RCP8.5 emission scenarios for three time-slices: 1979–2005, 2026–2045 and 2080–2100.

#### JRC: Multiple-model, multiple-scenario ensemble

Mentaschi *et al*.^20^ (hereafter JRC) developed a global wave climate projection dataset using 3-hourly surface wind forcing from six CMIP5 models to drive a global WW3 model at 1.5° grid resolution. The WW3 model was set up using the ST4 source-term physics with no sea-ice forcing fields. The simulations were conducted between 1970–2100 under emission scenarios RCP4.5 and RCP8.5.

#### USGS: Multiple-model, multiple-scenario ensemble

Li *et al*.^21^ (hereafter USGS) used 3-hourly surface winds (no sea-ice concentration) simulated by four CMIP5 GCMs to generate an ensemble of wave conditions for a recent historical time-period (1976–2005) and projections for the middle and end of the 21st century for 2 forcing scenarios (RCP4.5 and RCP 8.5). The wave fields were simulated by the wave model WW3, applied globally at 1 × 1.25° grid resolution.

#### NOC: Single-model, multi-scenario ensemble

Bricheno and Wolf^22^ (hereafter NOC) developed a global wave climate projection for RCP4.5 and RCP8.5 scenarios, using surface wind forcing fields from EC-EARTH and daily sea-ice concentration to drive a global WW3 wave model (using the ST4 source-term physics). The global simulation was conducted at ~0.7 × 0.5° between 1970–2100.

#### ECCC (d): Multiple-model, single-scenario ensemble

Casas-Prat *et al*.^23^ (hereafter ECCC(d)) developed a global wave climate projection dataset at 1° grid resolution (refined to 0.5° nearshore). The simulations were conducted using the WW3 model using the ST4 source-term physics, forced by 3-hourly surface winds and daily sea-ice fields taken from the RCP8.5 emissions scenario simulations by five CMIP5 climate models. Simulations were conducted for two time-slices: 1979–2005 and 2081–2100.

#### IHE-DELFT: Single-model, single-scenario multiple-run ensemble

Semedo *et al*.^24^ (hereafter IHE-DELFT) developed a dataset of global wave climate projections using the WAM4.5 model at a 1° spatial resolution forced by surface wind fields and sea-ice concentration from seven different EC-EARTH realizations under the RCP8.5 emissions scenario. The WAM model was set up with default ST3 source-term physics and the simulation period spanned from 1979–2100 continuously.

#### LBNL: Single-model, single-scenario ensemble

Timmermans *et al*.^25^ (hereafter LBNL) developed a high-resolution global wave climate projection using monthly sea-ice fields and 3-hourly surface winds taken from the Community Atmospheric Model (or ‘CAM5’), the atmospheric model of the NCAR Community Earth System Model at 0.25° horizontal resolution. These surface wind fields were used to drive a global WW3 model (using ST4 source-term physics) between 1995–2005. Four simulations were performed using the high-resolution wind fields each initialized with a different microscopically perturbed atmospheric state. Future wave conditions were generated using the high-resolution 0.25° CAM5 wind forcing for RCP8.5 between 2081–2100 using observed SST + 2 °C.

#### KU: Single-model, multiple-scenario ensemble

Shimura *et al*.^26^ (hereafter KU) developed an ensemble of global wave climate projections using the WW3 model forced by 6-hourly surface winds (and monthly sea-ice forcing) at 0.5625° horizontal resolution from the high-resolution atmospheric MRI-AGCM3.2 H model. The WW3 model was setup using ST4 source-term physics. The forcing of MRI-AGCM were four future SST conditions derived from CMIP5 GCMs under the RCP8.5 emissions scenario. Simulations were conducted for two time-slices: 1979–2005 and 2079–2100.

### CMIP5 GCM-forced statistical global simulations

#### IHC: Multiple-model, multiple-scenario ensemble

Camus *et al*.^27^ (hereafter IHC) developed a global wave projection dataset at 1° grid resolution on the basis of a weather-type statistical downscaling method. They used daily SLP fields as predictor from thirty CMIP5 climate models and a reference wave hindcast ‘Global Ocean Wave’ (GOW2.0) as predictand observations. A regression-guided clustering method based on linear regression and *k*-mean clustering was performed at each wave grid site of GOW2.0, from which estimates of average *H*_*S*_ and *T*_*m*_ were obtained for each weather type (WT). The wave climate projections were estimated from the future probability of WTs and the mean value of the variables associated with each WT at each wave grid node. The CFSR (Climate Forecast System Reanalysis) and GOW2 data from 1970–2015 were used in the training of the statistical relationship by comparing estimations of monthly wave parameters obtained using the statistical approach and from the time series of GOW2.0. To diminish GCM biases, the SLP data were adjusted such that they have the same climatological average and standard deviation as the CFSR SLP dataset, used as proxy for observations over 1975–2005. The simulations were performed for two time-slices: 1975–2005 and 2010–2100 (under emissions scenarios RCP4.5 and RCP8.5).

#### ECCC (s): Multiple-model, multiple-scenario ensemble

Wang *et al*.^28^ (hereafter ECCC(s)) developed a global dataset of statistical wave projections using a multivariate regression model with lagged dependent variable to represent a SLP-*H*_*S*_ (mean sea level pressure and significant wave height) relationship. ECMWF’s ERA-interim data was used to calibrate the statistical relationship between predictand *H*_*S*_ and its SLP-based predictors. To reduce biases, the CMIP5 simulated SLP data fields were adjusted such that they have the same climatological mean and standard deviation as the ERA-Interim SLP data (used as proxy for observations for 1981–2000). The time series of 6-hourly SLP-based predictors obtained from the RCP4.5 and RCP8.5 scenarios simulations by twenty CMIP5 climate models were input to the calibrated statistical model to make projections of 6-hourly *H*_*S*_ over a 150-year period from 1950–2100 under both scenarios.

### Data processing framework

The COWCLIP experimental protocol was defined to provide a systematic, community framework and infrastructure to support validation, intercomparison, documentation and access for global (and eventually regional) wave climate projections forced from CMIP atmospheric datasets. Inconsistency between data (due to different historical and future time-slices, emission scenarios and variables) has been a key factor precluding our ability to move forward.

Based on this framework, we removed wind-wave parameter uncertainty by adopting a set of wave variables - significant wave height (*H*_*S*_), mean wave period (*T*_*m*_) and mean wave direction (*θ*_*m*_) - from which a standard set of wave statistics was obtained (across annual, seasonal and monthly time-frame resolutions) in a consistent manner (Table 2)^31,32^. This is explained below in Data Generation Method. The resulting data over three frequencies and three variables, capturing seven statistical measures (for *H*_*S*_ and *T*_*m*_, and two for *θ*_*m*_) and seven extremes statistics measures (for *H*_*S*_ annual), represents the entire dataset available for CMIP5-forced wave climate projection data. We note however that the USGS ensemble was not available to process with the COWCLIP code (section 2.3.1) - only annual and seasonal means and 99th percentile of *H*_*S*_ were accessible.

**Fig. 1:**
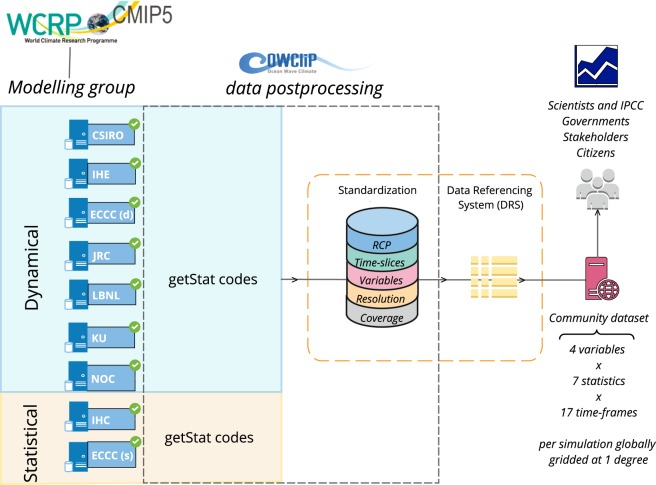
Flowchart of the COWCLIP2.0 experimental framework.

The flowchart of the experimental framework employed, and described below, is shown in Fig. 1.

### Data generation method

As part of the COWCLIP community framework, code was developed with programming language Fortran90 to ensure a consistent and precise computational data processing. The code comprises three functions (*getStat.f*, *getStatDir.f* and *getHsEx.f*) to calculate two standard sets of statistics, using sub-daily raw data from each standalone dataset^19–28^. During processing, the data was written to netCDF4 format. For information on access to (and guidelines for setup and usage of) the COWCLIP Fortran code, consult the Code Availability section.

### Standard statistics - getStat.f and getStatDir.f

The getStat.f code was designed to estimate statistics valid for scalar variables (*H*_*S*_, *T*_*m*_). The code was applied to each individual dataset separately^19–28^, enabling the calculation of seven wave statistics (mean, 10th, 50th, 90th, 95th, 99th percentiles, and maximum) for *H*_*S*_ and *T*_*m*_ calculated for monthly, seasonal and annual time-frame resolutions. The seasonal statistics were computed on default seasons defined as DJF, MAM, JJA and SON. The output netCDF files derived from each individual dataset retained all the relevant metadata of the input file and the coordinate variables/statistics. The names of the output files contained the time-frames of the statistics processed and the temporal resolution of the input data.

The *getStatDir.f* code is analogous to the *getStat.f*, but it was designed to calculate circular statistics meaningful for directional variables such as *θ*_*m*_. The code was applied to each standalone dataset^19–28^ (with available *θ*_*m*_) providing 2 circular statistics (mean and standard deviation) over the time-frames described above (Table 2).

### Extremes statistics - getHsEx.f

The *getHsEx.f* code was designed to calculate an ETCCDI set of extreme annual *Hs* indices from the sub-daily *Hs* input data^19–28^ (Table 1). The code was applied to each standalone dataset separately after concatenating the COWCLIP standard historical and future time-slices in a time sequence. A defined baseline period over 1986–2005 for relative statistics was adopted. The output netCDF files contained seven extreme statistics calculated annually (Table 3).

**Table 3:** Summary of the ETCDDI set of extreme significant wave height statistics included in the COWCLIP2.0 data set.

ETCCDI set of *H*_*S*_ statistics			
Statistics ID	Indicator name	Definition	Units
**via** ***getHsEx.f***			
HsRo	Rough wave days	Annual count of days when daily max *H*_*S*_ > 2.5 m	days
HsHi	High wave days	Annual count of days when daily max *H*_*S*_ > 6 m	days
fHsRo	Frequency of rough wave days	Annual percentage of days when daily max *H*_*S*_ > 2.5 m	%
fHsHi	Frequency of high wave days	Annual percentage of days when daily max *H*_*S*_ > 6 m	%
fHs10p^a^	Frequency of top decile wave days	Annual percentage of days when daily max *H*_*S*_ > 10th percentile of daily max *H*_*S*_ in the base period^a^	%
fHs90p^a^	Frequency of top decile wave days	Annual percentage of days when daily max *H*_*S*_ < 90th percentile of daily max *H*_*S*_ in the base period^a^	%
HHsDI^a^	Top decile wave spell duration indicator	Annual count of days with at least 2 consecutive days when daily max *H*_*S*_ > 90th percentile of daily max *H*_*S*_ in the base period^a^	days

^a^Relative statistics with base period 1980–2000 used for bootstrap procedure in relative statistics.

### Data assembly method

The netCDF files generated from each standalone dataset using the code described above, were used as a basis to build the collection of global wave climate projections following the standardization framework (see Fig. 1)^31,32^. In addition to removing parameter uncertainty, we also removed time-slice uncertainty between the processed datasets by using standardized historical (1979–2004) and future projection (2081–2100) time-slices. In terms of future emission scenarios, we processed data for two representative concentration pathways (RCPs)^37^: RCP4.5 and RCP8.5 defining a medium stabilization (+4.5 W/m^2^ forcing by the end of 21^st^ century) and a very high-emission scenario (+8.5 W/m^2^ forcing by the end of 21^st^ century), respectively.

Before assembling, each independent netCDF file underwent a quality-control analysis. The relevant statistics were extracted from each file (i.e. derived from each standalone dataset). The data compliant with the COWCLIP standard time-slices for simulation (for each frequency resolution), was extracted, and then converted to a global grid at 1° spatial resolution. For consistency, a mask was applied to exclude areas that are not captured by the full ensemble set of simulations (e.g. some simulations did not consider particular enclosed/semi-enclosed areas and others did not archive model outputs across regions with latitudes >60°N or S). After the regridding process, a shoreline dataset was imposed on the full set of wave simulations to ensure consistency between all the gridded data at the shoreline. The resultant data is therefore temporally and spatially consistent, without ‘undesirable’ uncertainties that previously hampered intercomparison analysis. Users seeking particular simulations (i.e., original simulated data developed by a specific climate modelling group) can be obtained with the individual modelling groups or through a request via the COWCLIP portal (data accessibility).

## Data Records

The full archived dataset^38^ comprising the different statistics described (consult the Data Generation Method) can be accessed through a *Scientific Data* recommended data repository: *Australian Ocean Data Network* (AODN) at DOI: 10.26198/5d91a9d00d60d.

The data set in total comprises 1372 files, with a total volume of 144 GB. The data is structured to mimic the DRS used for CMIP (and related data sets) and was specifically based on the DRS of the Coordinated Regional Downscaling Experiment (CORDEX)^39^ (as described in the CORDEX archive design: https://www.cordex.org/publications/report-and-document-archives/). This means a consistent directory structure and file naming convention is employed. Some wave modelling groups performed analysis across ensemble members within a GCM defined differently to the ‘r1i1p1’ definitions used within CMIP. Where this has occurred, the value for ‘ensemble’ in the DRS will take values relevant to that climate modelling group rather than standard CMIP5 values. The DRS adopted for the global COWCLIP2.0 dataset is as follows:

### Directories

*global/*<*modelling_centre*>*/*<*GCM*>/<*experiment>*/*ensemble*>*/*<*region*>/<*version*>/<*frequency*>*/*<*variable*>

### Filenames

<*variable*>_<*region*>_<*modelling_centre*>_<*GCM*>_<*experiment*>_<*ensemble*>*_*<*frequency*>_<*start_date*>-<*end_date>*.*nc*

Where <*region*> takes value “*glob*” and version is given in the form “vYYYYMM” (year/month). The Earth System Grid Federation convention is that files contain only one variable, however as we have produced three standard wave variables with two or seven statistical measures for each, as well as extremes statistics for annual *Hs* the files use <variable> values Hs, Tm, Dm, and HsEx, and each file contains multiple variables describing the statistics for that wave variable.

The data were made CF compliant by ensuring the ‘standard_name’ field was not erroneously used, variable ‘long_name’ was defined consistent with the Fortan90 code and units applied. No value for ‘_FillValue’ was provided and thus this has been omitted. Recommended global attributes are defined and included, drawing from the COWCLIP metadata table (Table 1) - which enable some additional compliance with the ACDD metadata standard.

Note that although every effort was made to ensure data adhered to both the CF and ACDD metadata conventions, the files are not strictly CF-compliant in time dimension - which uses units “years since” and “months since” the reference date. This is not advised by the CF convention since these values are ambiguous and depend on the calendar used. As the input data comes from CMIP5 models which use a variety of calendars and this information is not captured in the data generated by the *getStat* scripts, retrospectively applying calendar definitions was deemed to be less appropriate than using the more generic time definition, which is in line with the data produced by *getStat*.

## Technical Validation

All contributing datasets have undergone previous validation, with each individual study providing a model-skill assessment of developed GCM-forced global wave simulations against waverider buoy observations, and/or wave hindcasts/reanalysis, as reference^19–28^. Comparison of model-skill between all simulations relative to two well-validated historical datasets have also been conducted^34^, allowing an intercomparison of all simulated wave data under a common reference dataset.

**Fig. 2:**
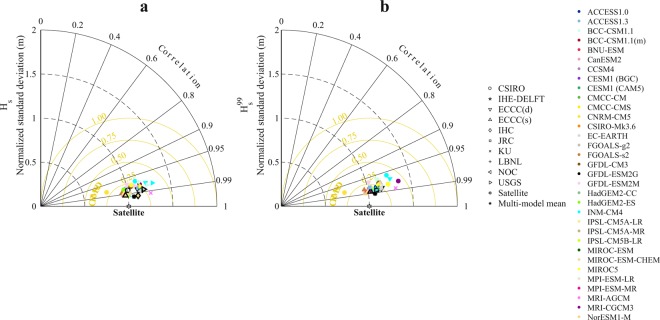
Taylor diagram for annual mean of *Hs* (**a**) and *H*_*s*_^99^ (**b**) of all global ocean region relative to the Satellite data over the period 1991–2017. The metrics shown are the spatial correlation (SC), normalized standard deviation (NSD) (given by *σ*_*sim*_/*σ*_*obs*_ derived from a specific simulation and the satellite dataset^40^) and the centred-root-mean-square (CRMSD) difference. The SC is shown by the azimuthal angle, the normalized standard deviation is shown by the radial distance from the origin (i.e., satellite data) and the CRMSD is shown by the distance from the origin (the yellow lines). Each colour denotes a specific model forcing and each symbol a specific modelling group. The symbols with black outline denote the ensemble mean of each study group when suitable and the asterisk to the full multi-member ensemble mean.

**Fig. 3:**
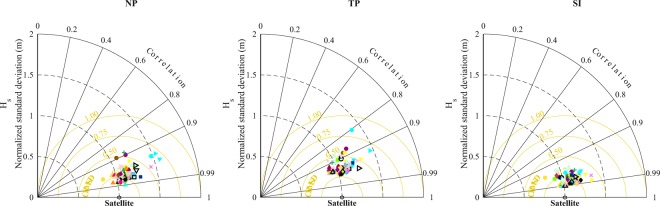
Taylor diagram for annual mean of *Hs* in 3-sub regions (North Pacific Ocean, Tropical Pacific and South Indian Ocean) of global ocean relative to the satellite data over period 1991–2017, respectively. The metrics shown are the spatial correlation (SC), normalized standard deviation (NSD) (given by *σ*_*sim*_/*σ*_*obs*_ derived from a given simulation and the satellite dataset^40^) and centred-root-mean-square (CRMSD) difference. The SC is shown by the azimuthal angle, the normalized standard deviation is shown by the radial distance from the origin (satellite data) and the CRMSD is shown by the distance from the origin (the yellow lines). Legend as per legend of Fig. 2.

The data produced for publication was verified to be numerically unchanged between the submitted netCDF, intermediate Matlab matrix, and final netCDF files. Comparison of the GCM-forced global wave simulations against satellite altimetry data^40^ (between 1991–2017). Note that climate models are not constrained to reproduce the timing of natural climate variability in the ‘observational record’, and consequently, our climate model-driven wave simulations are not in phase with observations. Hence, we can test the performance of the climatology (distribution) of model vs altimeter wave heights only; Figs. 2 and 3 are examples of skill analysis that have been previously done with respect to satellite measurements.

### Usage notes

The data is published via the *Australian Ocean Data Network* (AODN). The metadata record is available via GeoNetwork at ‘DOI’: 10.26198/5d91a9d00d60d. The dataset is accessible via the AODN THREDDS server (netCDF files) and can be accessed remotely using the OPeNDAP protocol at: http://thredds.aodn.org.au/thredds/catalog/CSIRO/Climatology/COWCLIP2/catalog.html. OPeNDAP is a protocol that allows netCDF files to be accessed from a remote server as though they were local on the file system. It is an effective mechanism to remotely subset files to extract only an area or time period of interest. This reduces the need for data replication and download. OPeNDAP file access is supported through most tools which permit analysis of the netCDF data files, including MATLAB, R, Python, ArcGIS and many others.

Due to the ambiguous nature of the time dimension defined without a calendar attribute, these files may display unexpected timestamps when read with some tools. We would advise the data consumer that use of this data with python’s Iris library or other libraries which depend on CF-compliance of the time dimension may be problematic.

## Supplementary information

Supplementary Table 1

## Data Availability

**Fortran code:** ***getStat.f***, ***getStatDir.f***, ***getHsEx.f*** The Fortran code developed to derive the COWCLIP statistics can be requested via the COWCLIP website (https://cowclip.org/data-access). The code - as described in the Data Generation Method section, consists of a set of commands (*getStat.f, getStatDir.f and getHsEx.f*) which can be compiled with a Fortran compiler, linked against netCDF4 and HDF5 libraries. The documentation for setup, usage and requirements for the code is described within the technical reports^30–32^ that complement this manuscript. These commands can be executed by COWCLIP contributors to generate the set of wave statistics from their raw simulations. With the specific purpose of sharing in an open data format, and adhering to relevant data standards, the processed data is given in netCDF format, the global metadata attributes from the submitted netCDF data recorded, and additional information added where possible to ensure both CF Conventions and Attribute Convention for Dataset Discovery (‘ACDD’) standards compliance. **Python code:** ***COWCLIP_stats_mat2nc.py*** **and** ***COWCLIP_extremes_mat2nc.py*** The Python commands developed to produce the final standardised netCDF files (which comprise this data publication) are available in the COWCLIP website (https://cowclip.org/data-access). The code is written in Python 3 as well as standard python modules, depends on numpy, pandas, scipy, tables and netCDF4 python modules. Both python scripts require setting of the descriptive metadata location (path to file COWCLIP-GlobalProj-Metadata-merged.xlsx, structured to be readily usable with python’s pandas library), and the location of the Matlab matrix (.mat) and script (.m) files for the standardised data. The python scripts take as command line arguments the climate modelling group (e.g., ‘LBNL’) and time-slice simulation period (i.e., ‘Historical’ or ‘Future’). They produce, where possible, CF and ACDD standards-compliant output files in a Data Reference Syntax (DRS) structure akin to that used in CMIP and CORDEX modelling projects.
