# Supplementary material for: A global ensemble of ocean wave climate projections from CMIP5-driven models
Source: Sci Data. 2020 Mar 27;7:105. doi: 10.1038/s41597-020-0446-2 (PMC7101334; doi:10.1038/s41597-020-0446-2)
Supplement: Supplementary file 1 — Supplementary Table 1 [file 41597_2020_446_MOESM1_ESM.docx]

| **Supplementary Table S1** - Summary of the wave contributions to the COWCLIP2.0 intercomparison data set. The emission scenarios (RCP pathways) and climate-model forcing used by each group to derive wave-climate projections is shown. The wave climate ensemble members provided by each contributing group are coloured. *Please note that the MRI-AGCM, MIROC5, MIROC-ESM and MIROC-ESM-CHEM CMIP5 data are released under non-commercial terms of use. As such all data presented here are available under a CC-BY-SA licence, with the exception of data derived from the MIROC models which is released under a CC-BY-NC licence. | | | | | | | | | | | | |
| --- | --- | --- | --- | --- | --- | --- | --- | --- | --- | --- | --- | --- |
| Research centre | CSIRO^19^ | JRC^20^ | USGS^21^ | NOC^22^ | ECCC (d)^23^ | IHE^26^ | LBNL^27^ | KU^28^ | IHC^29^ | | ECCC (s)^30^ | |
| Country | Australia | EU | US | UK | Canada | Netherlands | US | Japan | Spain | | Canada | |
| Emission scenario | RCP4.5/8.5 | RCP4.5/8.5 | RCP4.5/8.5 | RCP4.5/8.5 | RCP8.5 | RCP8.5 | RCP8.5 | RCP8.5 | RCP4.5/8.5 | | RCP4.5/8.5 | |
| CMIP5 GCM(s) forcing used | | | | | | | | | | | | |
| ACCESS1.0 |  |  |  |  |  |  |  |  |  | |  | |
| ACCESS1.3 |  |  |  |  |  |  |  |  |  | |  | |
| BCC-CESM1.1 |  |  |  |  |  |  |  |  |  | |  | |
| BCC-CESM1.1(m) |  |  |  |  |  |  |  |  |  | |  | |
| BNU-ESM |  |  |  |  |  |  |  |  |  | |  | |
| CanESM2 |  |  |  |  |  |  |  |  |  | |  | |
| CESM1 (BGC) |  |  |  |  |  |  |  |  |  | |  | |
| CESM1 (CAM5) |  |  |  |  |  |  |  |  |  | |  | |
| CCSM4 |  |  |  |  |  |  |  |  |  | | *r6i1p1*^a^ | |
| CMCC-CM |  |  |  |  |  |  |  |  |  | |  | |
| CMCC-CMS |  |  |  |  |  |  |  |  |  | |  | |
| CNRM-CM5 |  |  |  |  |  |  |  |  |  | |  | |
| CSIRO-Mk3.6 |  |  |  |  |  |  |  |  |  | |  | |
| EC-EARTH |  | *r8i1p1*^a^ |  | *r12i1p1*^a^ | *r2i1p1*^a^ |  |  |  |  | | *r2i1p1*^a^ |  |
| FGOALS-s2 |  |  |  |  |  |  |  |  |  | |  | |
| FGOALS-g2 |  |  |  |  |  |  |  |  |  | |  | |
| GFDL-CM3 |  |  |  |  |  |  |  |  |  | |  | |
| GFDL-ESM2G |  |  |  |  |  |  |  |  |  | |  | |
| GFDL-ESM2M |  |  |  |  |  |  |  |  |  | |  | |
| HadGEM2-CC |  |  |  |  |  |  |  |  |  | |  | |
| HadGEM2-ES |  |  |  |  |  |  |  |  |  |  |  | |
| INMCM4 |  |  |  |  |  |  |  |  |  | |  | |
| IPSL-CM5A-LR |  |  |  |  |  |  |  |  |  | |  | |
| IPSL-CM5A-MR |  |  |  |  |  |  |  |  |  | |  | |
| IPSL-CM5B-LR |  |  |  |  |  |  |  |  |  | |  | |
| MIROC-ESM* |  |  |  |  |  |  |  |  | * | | * | |
| MIROC-ESM-CHEM* |  |  |  |  |  |  |  |  | * | | * | |
| MIROC5* | * |  | * |  | * |  |  |  |  | |  | |
| MPI-ESM-LR |  |  |  |  |  |  |  |  |  | |  | |
| MPI-ESM-MR |  |  |  |  |  |  |  |  |  | |  | |
| MRI-CGCM3 |  |  |  |  |  |  |  |  |  | |  | |
| NorESM1-M |  |  |  |  |  |  |  |  |  | |  | |
| CMIP5-based/observed SST forcing used | | | | | | | | | | | | |
| CAM5-AGCM (SST^b^ +2°) |  |  |  |  |  |  |  |  |  | |  | |
| MRI-AGCM-SST0^c^ |  |  |  |  |  |  |  |  |  | |  | |
| MRI-AGCM-SST1^c^ |  |  |  |  |  |  |  |  |  | |  | |
| MRI-AGCM-SST2^c^ |  |  |  |  |  |  |  |  |  | |  | |
| MRI-AGCM-SST3^c^ |  |  |  |  |  |  |  |  |  | |  | |

^a^model run number as per CMIP5 syntax (*r* for realization, *i* for initialisation and *p* for physics, followed by integer). All other runs used ensemble member *r1i1p1*.

^b^observed SST obtained from the HadISST1-based data set were used to force the atmospheric model CAM5.

^c^SST0 to SST3 correspond to four different SST future change patterns derived from CMIP5 GCM models to force the atmospheric model MRI-AGCM^32^.
